# Antidote for Cupreous Salts

**Published:** 1852-09

**Authors:** 


*Antidote for Cupreous Salts.*—M. Roncha, of Strasburg, states that calcined magnesia completely checks the symptoms occasioned by poisoning with sulphate of copper, if taken quickly after the copper salt; but it requires a large quantity of magnesia, eight times the weight of the sulphate of copper, to neutralise the effects of this salt. It is stated as probable, that magnesia will prove one of the best antidotes to cupreous salts, a statement in which at present we should be sorry to express our acquiescence.—*Ibid.*

